# Pre-clinical activity of the oral DNA-PK inhibitor, peposertib (M3814), combined with radiation in xenograft models of cervical cancer

**DOI:** 10.1038/s41598-021-04618-5

**Published:** 2022-01-19

**Authors:** Sushmita B. Gordhandas, Beryl Manning-Geist, Christina Henson, Gopa Iyer, Ginger J. Gardner, Yukio Sonoda, Kathleen N. Moore, Carol Aghajanian, M. Herman Chui, Rachel N. Grisham

**Affiliations:** 1 Department of Surgery, Memorial Sloan Kettering Cancer Center, New York, NY USA; 2 University of Oklahoma Health Sciences Center, Oklahoma City, OK USA; 3 Department of Medicine, Memorial Sloan Kettering Cancer Center, New York, NY USA; 4 Weill Cornell Medical College, New York, NY USA; 5 Department of Pathology, Memorial Sloan Kettering Cancer Center, New York, NY USA

**Keywords:** Cancer, Cancer therapy, Gynaecological cancer

## Abstract

DNA-dependent protein kinase (DNA-PK) plays a crucial role in repair of DNA double-strand breaks by facilitating non-homologous end-joining. Inhibitors of DNA-PK have the potential to block DNA repair and enhance DNA-damaging agents. Peposertib (M3814) is a DNA-PK inhibitor that has shown preclinical activity in combination with DNA-damaging agents, including ionizing radiation (IR) and topoisomerase II inhibitors. Here we evaluated the activity of peposertib (M3814) in combination with radiation in a mouse xenograft model of HPV-associated cervical cancer. Athymic nude female mice with established tumors derived from HeLa cells injected into the flank were treated with vehicle alone (n = 3), IR alone (n = 4), and peposertib (M38814) in combination with IR (M3814 + IR; n = 4). While IR alone was associated with a trend towards decreased tumor volume compared with untreated, only the M3814 + IR treatment arm was associated with consistent and significant reduction in tumor burden, which correlated with higher levels of γ-H2AX in tumor cells, a marker of double-strand DNA breaks. Our data support further clinical evaluation of the combination of peposertib (M38814) and IR in cervical cancer.

## Introduction

DNA damage is inherent in the process of DNA replication, and repair of this damage is essential for normal cell function. Cells have evolved mechanisms for DNA repair to maintain genomic integrity. One important type of DNA damage is double-strand breaks (DSB). DSBs can be induced from oxidative stress, oncogene induced transcription stress, or therapeutic treatment of cancer with chemotherapy or radiation^1^. DSBs have lethal consequences for cells and organisms if left unrepaired^2^. There are two main pathways to repair DSBs: homologous recombination-guided repair (HR) and non-homologous end joining (NHEJ).

DNA-dependent protein kinase (DNA-PK) has been shown to play a critical role in facilitating NHEJ^3–6^.The DNA-PK complex consists of catalytic serine/threonine protein kinases and two heterodimeric subunits (KU80 and KU70). Elevated DNA-PK expression has been associated with poor cancer-specific survival in ovarian cancer patients^7^. Blocking DSB repair increases susceptibility to DNA-damaging agents through accumulation of damage and eventually cell death^8,9^. Cancer therapies targeting the repair mechanisms of DSBs have led to improved outcomes, such as poly (ADP-ribose) polymerase (PARP) inhibition in ovarian cancer^10–12^.

Peposertib (M3814) is a potent, selective, and orally bioavailable DNA-PK inhibitor^2^. Preclinical studies of peposertib (M3814) in combination with ionizing radiation (IR) are compelling^2,13^. Zenke and colleagues evaluated the IC50 and EC50 of cervical cancer cell lines C33A, CASKI and HeLa for treatment with peposertib (M38814) alone and in combination with 3 Gy IR. Growth viability was assessed and demonstrated sensitivity of all 3 cervical cancer cell lines to peposertib (M3814) in combination with IR and evidence of synergistic affect as calculated by Bliss independence in the HeLa and C33A cell lines^2^. In xenograft models of human cancers (squamous cell head and neck, and non-small cell lung cancer), oral administration of peposertib (M38814) strongly potentiated the anti-tumor activity of IR leading to complete tumor regression at nontoxic doses^2^. Additional mouse models have demonstrated that peposertib (M38814) in combination with IR also has activity in colon and pancreatic cancer^13^. These studies provide evidence that inhibition of DNA-PK sensitizes cancer cells to DSBs induced by IR.

Targeting DNA damage response pathways is a potentially effective strategy for treatment of HPV-associated cervical cancer. HPV-associated tumors have overexpression of E6/E7 oncoproteins that promote tumor growth and increase DNA damage including accumulation of DSBs^14^. IR is already essential in upfront treatment of many early and late stage cervical cancers, but in the recurrent setting there are few effective systemic therapies, and data on the efficacy of IR alone are limited^15,16^. In this study, we aimed to determine if the oral DNA-PK inhibitor peposertib (M3814) could enhance the efficacy of IR in models of cervical cancer. We hypothesize that the combination of peposertib (M3814) and IR will provide cervical cancer patients with new treatment options that are both efficacious and tolerable.

## Results

### DNA-PK inhibitor peposertib (M3814) enhances activity of radiation in vivo

**Figure 1:**
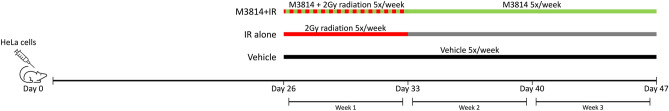
Three million HeLa cells were injected subcutaneously into the flank of eleven 11 athymic nude female mice. The animals were separated into three groups: vehicle (n = 3), IR alone (n = 4), and M3814 + IR (n = 4). Treatment started 26 days after injection. The vehicle group received M3814 vehicle control by oral gavage once daily 5 days per week for weeks one to three. The IR alone group received 2 Gy radiation 5 days per week for week one. The combined M3814 + IR group received peposertib (M38814) 50 mg/kg by oral gavage once daily 5 days per week for weeks 1–3, and 2 Gy radiation 5 days per week for week one. Radiation was administered 60 min after M3814 drug administration.

To test the efficacy of peposertib (M3814) in combination with IR we performed in vivo xenograft studies using the HeLa cell line, as a model of HPV-associated cervical cancer (HeLa). HeLa cells were implanted in the flank of 11 athymic nude female mice and treatment was started at 26 days after cell implantation (Fig. 1). Tumor volume ranged from 130 to 133 mm^3^ at the start of treatment. Mice were separated into three treatment groups: vehicle alone (n = 3), IR alone (n = 4), and peposertib (M38814) in combination with IR (M3814 + IR) (n = 4) (Fig. 1).

**Figure 2:**
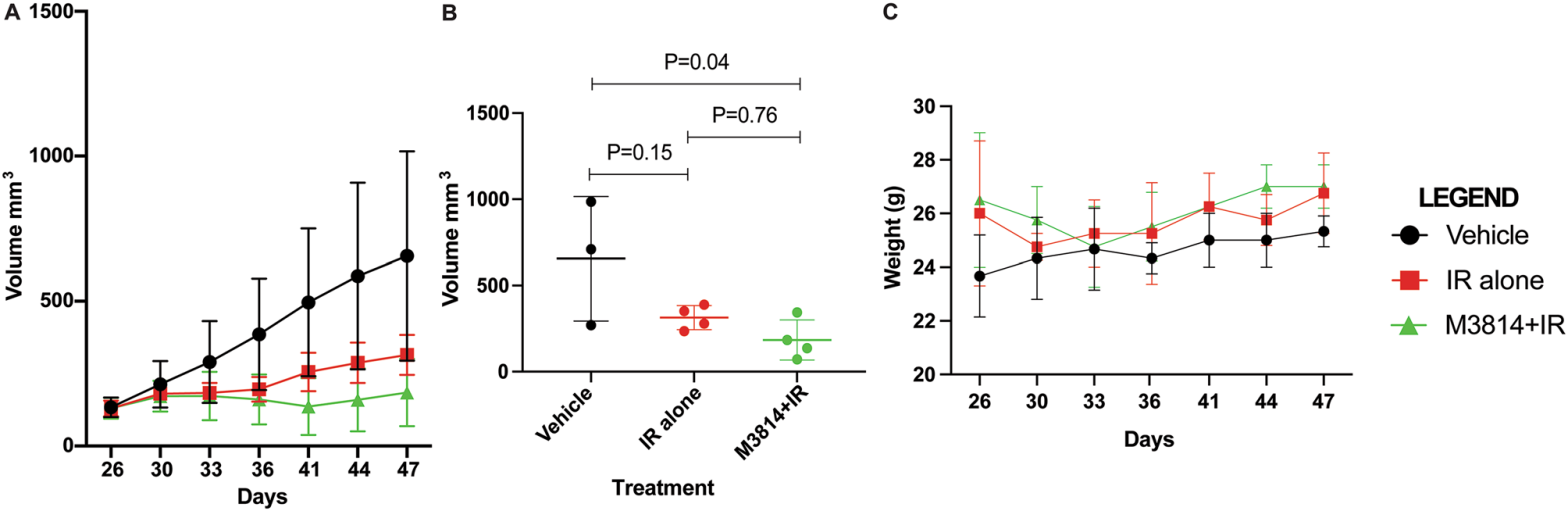
Xenograft experiments were performed with HeLa cell lines in athymic nude mice. Treatment was started 26 days after HeLa cell implantation. Three groups were investigated: vehicle alone (n = 3), IR alone (n = 4), or M3814 in combination with IR (n = 4). Tumor volume was measured twice weekly. (**A**) shows mean tumor volume of each group over the course of treatment. (**B**) shows average tumor volume at treatment endpoint (day 47), *t* tests were done to compare the treatment groups. (**C**) shows mouse weights during the course of treatment.

Mice treated with M3814 + IR had slower growth of tumor over time compared with IR alone or vehicle (Fig. 2A). At treatment endpoint (day 47) mean tumor volumes in the vehicle alone, IR alone, and M3814 + IR groups were 655 mm^3^ (SD 360.7), 314 mm^3^ (SD 69.5), and 184 mm^3^ (SD 116.4), respectively (Fig. 2B). Mean tumor volume at end of treatment in IR alone was not statistically different from that of vehicle alone (*p* = 0.12) or M3814 + IR (*p* = 0.10). Vehicle alone had a significantly higher volume of tumor at end of treatment in comparison with M3814 + IR (*p* = 0.05). Body weights remained stable throughout the experiment (Fig. 2C).

**Figure 3:**
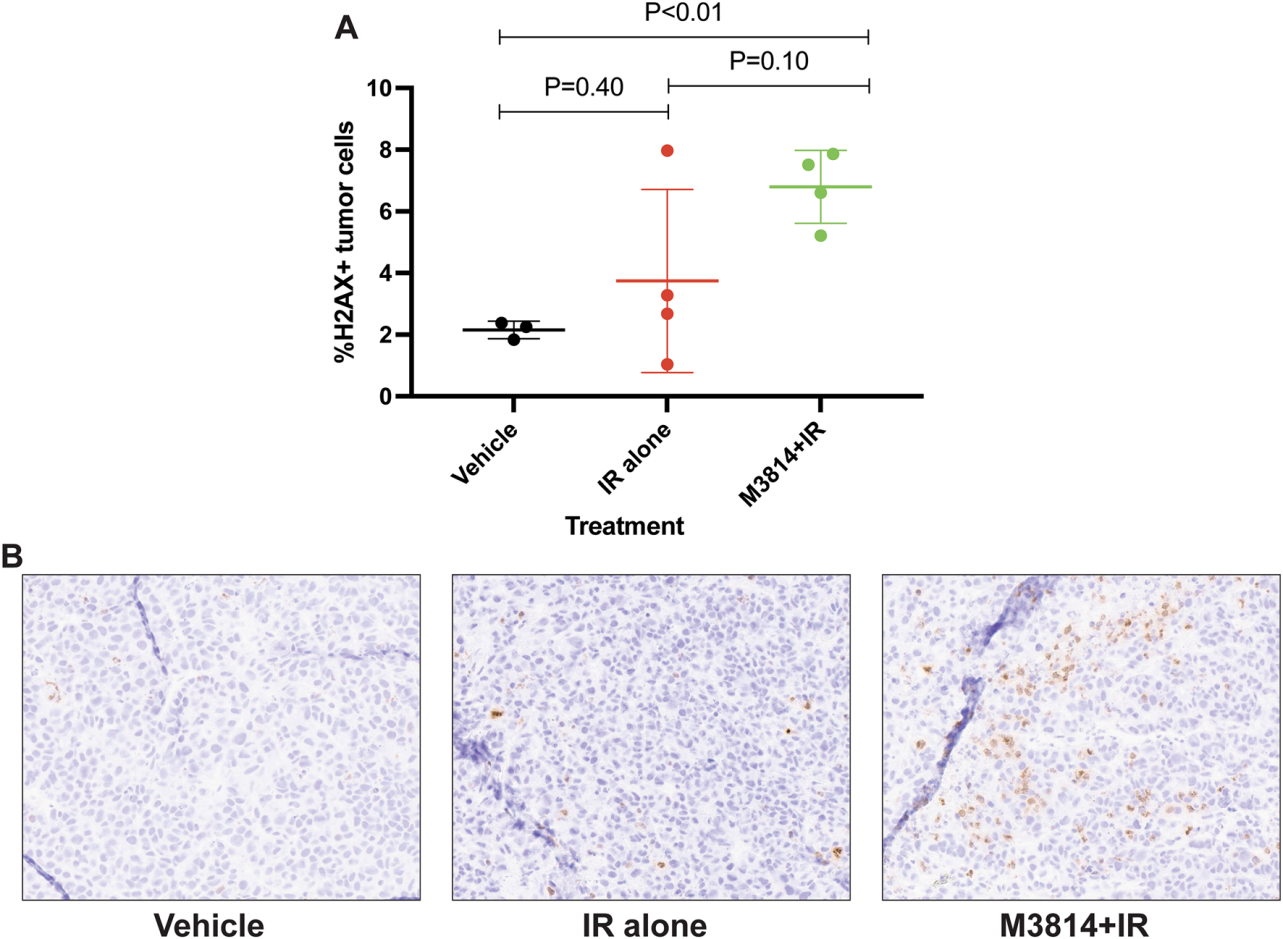
(**A**) γ-H2AX antibody staining was performed in formalin-fixed tumor tissue for each group at the completion of treatment. DNA damage was significantly increased in the M3814 + IR group in comparison to vehicle alone (*p* < 0.01). (**B**) Micrographs of γ-H2AX antibody staining in each group.

To evaluate DNA damage, immunohistochemical analysis of γ-H2AX was performed on tumors excised from all sacrificed mice at the completion of treatment. DNA damage was significantly increased in the M3814 + IR group in comparison with vehicle alone (*p* < 0.01; Fig. 3A). Comparisons of γ-H2AX staining in IR alone versus vehicle alone and M3814 + IR were not statistically significant (*p* = 0.4 and 0.1, respectively). Representative micrographs of γ-H2AX antibody staining in each group are displayed in Fig. 3B.

## Discussion

Standard of care treatment for newly diagnosed, non-metastatic cervix cancer is well-established, but treatment options for recurrent cervical cancer are limited. Following systemic treatment with taxane and platinum, post-progression data from GOG 0240 showed a median overall survival of only 7.1 and 8.4 months in the non-bevacizumab and bevacizumab arms, respectively^17^. Second-line cytotoxic monotherapy also has low overall response rates: topotecan (12.5%), vinorelbine (13.7%) and pemetrexed (15%)^18^. The current standard of care includes pembrolizumab for women with programmed death-ligand 1 (PD-L1) positive tumors. PD-L1 is expressed in approximately 50% of squamous cell cervical carcinomas, and 14% of cervical adenocarcinomas^19^.

Keynote-158 reported an overall response rate of 14.6% and duration of response of 9 months with pembrolizumab in a single arm cohort^20^. A subsequent randomized phase 3 trial of the anti PD-1 antibody cemiplimab versus investigator choice chemotherapy confirmed the superiority of immune checkpoint inhibitors in this setting with an overall response rate of 17.6%, compared with 6.7% for cemiplimab versus chemotherapy, respectively. More importantly, overall survival was improved with a hazard ratio of 0.69 (95% CI 0.56–0.84; *p* < 0.001) in favor of cemiplimab. Median overall survival was 12.0 versus 8.5 months^21^. While PD-L1 targeted therapies are a welcome advance in treatment for these often-young patients, there is a high unmet need for novel therapies with more efficacy and broader applicability.

Radiation therapy (RT) is a non-surgical option for local recurrence of cervical cancer in women who have not previously been treated with RT, or with operable disease who opt not to proceed with pelvic exenteration. Data on the efficacy of RT in the recurrent setting is limited. In a 2008 paper Haasbeek et al. described their single institution experience of high dose RT following a pelvic recurrence of cervical cancer (n = 35); the five- and ten-year survival rates were 43% and 33%, respectively, and pelvic control rates were 69% and 62%, respectively^16^. The amount of radiation must be considered carefully to reduce toxicity to normal tissue, particularly given that this area often has already been heavily irradiated^22^. Chemotherapy in combination with RT has shown improved response in cervical cancer, but combined treatment is often limited by hematologic toxicity and bone marrow suppression, especially in pre-treated patients. New combination therapy options for cervical cancer are needed to improve the efficacy of radiation and limit toxicity.

DNA-PK inhibitors have shown activity in combination with DNA damaging agents, highlighting their potential to improve the efficacy of RT. We studied the effect of peposertib (M3814), an oral DNA-PK inhibitor, in combination with IR in cervical cancer models. Prior studies have demonstrated that peposerib (M3814) has limited efficacy as a single agent in gynecologic cancer models, highlighting the importance of DNA damage in its mechanism of action^23^. The effect of peposerib (M3814) in combination with therapies that induce DNA damage was emphasized in our in vivo results. When compared with vehicle alone, the combination of M3814 + IR produced a more pronounced effect on tumor volume than IR alone (*p* = 0.04 and 0.15, respectively). These findings suggest aneffect of M3814 in the treatment of cervical cancer. Weights were stable throughout therapy, suggesting tolerability of the combined treatment (Fig. 2C).

In addition to measuring treatment response with tumor volume, γ-H2AX IHC staining was performed on all tumors at the completion of treatment. γ-H2AX staining is a marker of DNA double-strand breaks and can be used to monitor DNA damage after therapy^24^. The γ-H2AX staining results reflect the findings of treatment response measured by tumor volume; M3814 + IR had significantly increased DNA damage compared with vehicle alone (*p* < 0.01). All other comparisons were not statistically significant (Fig. 3A). The IR alone group includes one sample (Fig. 3A, animal no. 4) with increased γ-H2AX staining, this is likely due to technical factors. The γ-H2AX antibody has some non-specific staining and staining of cell debris which contributes to intrinsic variability in quantification. In addition to technical challenges, small sample size has limited our ability to find statistically significant differences and determine magnitude of effect. M3814 + IR had slower tumor growth and increased DNA damage compared with IR alone, but these comparisons were not statistically significant.

Our study adds to the pre-clinical literature in support of peposertib (M3814) in combination with DNA damaging agents. Clinical data from completed and ongoing trials show the safety of peposertib (M3814) in cancer patients. As of October 2020, a total of 137 subjects have been exposed to peposertib (M3814) in five clinical studies^25^. In a monotherapy dose escalation study (EMR100036-01), the maximum tolerated dose of M3814 was not reached up to the highest administered level, providing evidence for the tolerability of peposertib (M3814) for patients. The maximum tolerated dose in combination with RT has not been determined yet. The main adverse events of peposertib (M3814) were rash-related and clinically manageable.

Clinical trials of therapies targeting the DNA damage response pathway have shown promising results^26^. Our group has previously published on the activity of peposertib (M3814) in combination with topoisomerase II inhibitors, the combination delayed tumor growth when compared with monotherapy controls in murine grafts, and a phase IB clinical trial of peposertib (M3814) in combination with cytotoxic therapy is underway for recurrent ovarian cancer (NCT04092270). Based on the pre-clinical data presented in this paper, clinical trials for the combination of peposertib (M3814) with RT in cervical cancer are warranted. A phase I/II study of peposertib (M3814) with hypofractionated radiation followed by peposertib (M3814) with avelumab (PD-L1, checkpoint inhibitor) in recurrent cervical cancer is currently under development.

## Methods

All animal experiments were performed at Memorial Sloan Kettering’s Research Animal Resource Center and were carried out in accordance with relevant guidelines and regulations. The experiments were approved by the Memorial Sloan Kettering Institutional Animal Care and Use Committee (protocol # 04-03-009). All methods are reported in accordance with ARRIVE guidelines for the reporting of animal experiments.

Three million HeLa cells with Matrigel, were injected subcutaneously into a single flank of eleven 6- to 8-week old athymic nude female mice. The animals were separated into three groups: vehicle alone (n = 3), IR alone (n = 4), and peposertib (M38814) in combination with IR (M3814 + IR) (n = 4). Treatment was started 26 days after subcutaneous injection (Fig. 1).

The M3814 vehicle control was 0.5% Methocel™, 0.25% Tween20, 300 mM Na-Citrate Buffer, pH 2.5 by oral gavage, five days per week for weeks one to three. In the IR alone group, local 2 Gy radiation was administered five days per week for week one. In the combined M3814 + IR group, peposertib (M3814) was administered at 50 mg/kg by oral gavage once daily five days per week for weeks one to three, and irradiation was done 60 min after peposertib (M3814) drug administration (2 Gy 5 days per week for week one) (Fig. 1).

All mice were evaluated for weight and tumor volume on the same days twice per week starting 26 days after cell implantation. Tumor volume at end of treatment was compared for treatment with vehicle alone versus IR alone, IR alone versus M3814 + IR, and vehicle alone versus M3814 + IR using *t* tests (Prism for macOS version 9.2.0) (Fig. 2B). At completion of week three, all mice were sacrificed. Tumors were excised, formalin-fixed and paraffin embedded. Immunohistochemical staining was performed for γ-H2AX using a rabbit monoclonal antibody (Cell Signaling, phospho-histone H2AX, clone 20E3, Cat no. 9718, 1:200 dilution). Quantification of immunohistochemical staining was performed on digitally scanned slides using the Positive Cell Detection algorithm in QuPath software, to determine the percentage of γ-H2AX-postive tumor cells. At least 5000 tumor cells were analyzed per sample.

### Ethical approval and informed consent

All animal experiments were performed at Memorial Sloan Kettering’s Research Animal Resource Center and were carried out in accordance with relevant guidelines and regulations. All animal experiments were approved by the Memorial Sloan Kettering Institutional Animal Care and Use Committee (protocol # 04-03-009). All methods are reported in accordance with ARRIVE guidelines for the reporting of animal experiments.
